# Interactions between UCP2 SNPs and telomere length exist in the absence of diabetes or pre-diabetes

**DOI:** 10.1038/srep33147

**Published:** 2016-09-12

**Authors:** Yuling Zhou, David Simmons, Brett D. Hambly, Craig S. McLachlan

**Affiliations:** 1 University of New South Wales, Rural Clinical School, Sydney, Australia; 2 University of Melbourne, Rural Clinical School, Shepparton, Australia; 3 Western Sydney University, School of Medicine, Sydney, Australia; 4 University of Sydney, Discipline of Pathology and Bosch Institute, Sydney, Australia

## Abstract

Mitochondrial uncoupling protein 2 (UCP2) can affect oxidative stress levels. *UCP2* polymorphisms are associated with leukocyte telomere length (LTL) in Type 2 Diabetes, which also induces considerable background oxidative stress. The effects of *UCP2* polymorphisms on LTL in populations without diabetes have not been well described. Our aims are to evaluate the interaction between LTL and *UCP2* polymorphisms in 950 subjects without diabetes. The monochrome multiplex quantitative PCR method was used to measure relative LTL. Taqman SNP genotyping assay was applied to genotypes for *UCP2* rs659366 and rs660339. We found shorter LTL associated with increased age (*P *< 0.001) and triglyceride levels (*P *= 0.041). After adjustment for cardiovascular risk factors, rs659336 GG genotype carriers demonstrated a shorter LTL (1.257 ± 0.186), compared to GA carriers (1.288 ± 0.230, *P *= 0.022) and AA carriers (1.314 ± 0.253, *P *= 0.002). LTL was shorter in the CC rs660339 genotype (1.254 ± 0.187) compared to TT (1.297 ± 0.242, *P* = 0.007) and CT carriers (1.292 ± 0.229, *P* = 0.016). The T allele of rs660339 is associated with a longer LTL of approximately 0.04 compared to CC homozygotes. Thus, *UCP2* rs659366 A allele and rs660339 T allele are both related to longer LTL in subjects without diabetes, independent of cardiovascular risk factors.

Telomeres are thousands of tandem repeats of “TTAGGG” DNA sequence and associated proteins, ranging from a few to 15 kilobases in length, at the end of eukaryotic chromosomes, that maintain the replicative potential of somatic cells. Shortened telomeres are associated with physiological aging and oxidative stress^1^,^2^. It is well known that mitochondrial reactive oxygen species can lead to shortening of telomeres^3^,^4^. Less is known about the genetic control of mitochondrial ROS and its interactions with cellular aging, i.e. shorter telomeres.

It is not surprising that insulin resistance, metabolic syndrome and type 2 diabetes mellitus (T2DM) have been linked to telomere shortening in cross sectional population studies^5^,^6^,^7^. Both oxidative stress and shorter telomere length are risk factors for age-related diseases, such as T2DM^1^,^8^,^9^ and related cardiovascular disease in T2DM^10^,^11^.

Mitochondrial uncoupling protein-2 (UCP2) is a widely expressed inner mitochondrial membrane protein that influences the regulation of reactive oxygen species (ROS), inflammation, cell death and mitochondrial membrane potential^12^,^13^,^14^,^15^. ROS may activate UCPs and cause a mild bio-energetic uncoupling of mitochondrial function. Mild mitochondrial uncoupling via UCP2 acts as a potential “safety factor” by providing negative feedback to dampen down further mitochondrial ROS production^16^,^17^. For example, reduced functional UCP2 expression is associated with elevated levels of oxidative stress and inflammation in translational studies^18^,^19^.

The most reported *UCP2* nucleotide polymorphisms are rs659366 (-866G >A) in the promoter region and rs660339 (C to T transition, Ala55Val) in the exon 4. Both these polymorphisms have been associated with T2DM and related complications. Rs659366 occurs in a cis regulatory element controlling UCP2 mRNA expression in adipocytes *in vivo* and correlates with obesity^20^, UCP2 expression in beta cells^21^, insulin levels^22^, risk of coronary disease^23^ and low density lipoprotein cholesterol^24^. Rs660339 is associated with insulin levels^25^ and energy expenditure cost of excise^26^,^27^. However, there have been inconsistent results for an association with T2DM risk^28^,^29^.

To date, two studies have examined the relationship between *UCP2* genotypes and leukocyte telomere length (LTL). The first study^30^ examined the relationship between the genotypes for both rs659366 and rs660339 and LTL in a diabetic patient cohort. The presence of the rs659366 A allele was found to be associated with shorter LTL, while no association was seen with the rs660339 genotype. This paper did not report on any association within the control groups. The second study^31^ examined the rs659366 genotype in participants with and without diabetes, but did not see any association with LTL. Similarly, these investigators did not see any association within the control group. Thus, the important question concerning whether an association exists between these *UCP2* genotypes and LTL remains unanswered in those without insulin resistance and/or diabetes.

The role of genetic *UCP2* mitochondrial polymorphisms and their contribution to the pathogenesis of telomere shortening remains incompletely understood. Deciphering telomere length and the role of *UCP2* gene contributions requires a non-insulin resistant state to remove any influence of disease-related background oxidative stress and associated inflammation. Our aims are to determine genetically whether there is an interaction between underlying LTL and *UCP2* polymorphisms in the absence of diabetes and pre-diabetes. Sub studies will control for cardiovascular risk factors, which may promote inflammation and/or oxidative stress and thereby influence LTL.

## Results

### Baseline characteristics and UCP2 SNPs genotyping

The study population of 950 subjects older than 25 years was obtained from a largely (>95%, Australian Census 2011) Caucasian rural Australian community, with an average age of 50.2 ± 15.2 years and 59.6% female. For *UCP2* rs659366 100% of samples were genotyped successfully, and for *UCP2* rs660339 99.26% (943/950). Reference to the Hapmap database (release 28 Phase II + III, August 10, on NCBI B36 assembly) revealed that the A allele frequency for rs659366 and rs660339 with European ancestry, are 37% and 42%, respectively. Our data show a similar frequency of the A allele for rs659366 (37%) and the A allele for rs660339 (41%), respectively, which is expected given the high levels of European ancestry in our rural population (>95%). Genotype rs660339 deviates from the Hardy-Weinberg equilibrium (*P *= 0.002), whereas rs659366 (*P *= 0.172) does not.

### Baseline demographics, UCP2 and LTL

Baseline demographics and mean cardiovascular risk factors across genotype subgroups rs659366 and rs660339 revealed no significant differences, with the exception of triglyceride levels for rs660339 (Table 1). Across rs660339 genotypes, TT, CT and CC carriers, mean blood triglyceride levels were 1.52 ± 1.74 mmol/L, 1.27 ± 0.71 mmol/L and 1.35 ± 0.83 mmol/L, respectively (*P* = 0.028). As baseline cardiovascular risk factors show no difference between SNP genotype subgroups (apart from triglycerides), selection bias across samples was minimal.

We then explored known cardiovascular risk factors and demographic factors that influence telomere length (Table 2). As expected, chronological age was associated with shorter telomere length in our model (*P* < 0.001). The only cardiovascular risk factor that was associated with mean LTL was triglycerides levels (*P* = 0.041), while gender, smoking status, BMI, waist circumference, HDL and SBP did not have a significant interaction on LTL.

### Telomere Length and UCP2 SNPs genotype

**Figure 1:**
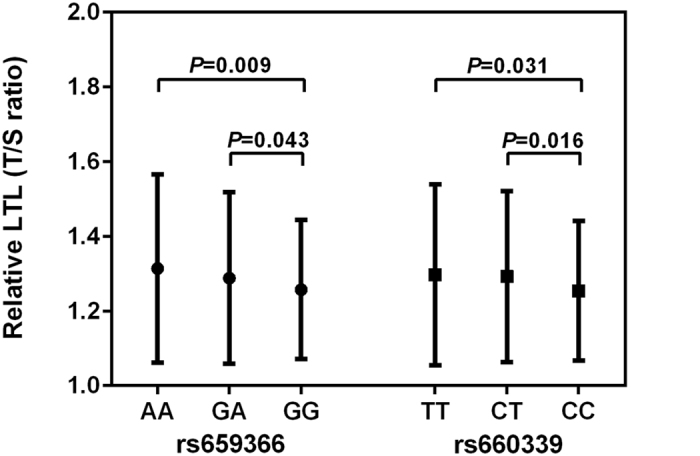
Comparison of relative telomere length for UCP2 SNPs rs659366 and rs660339. ANOVA was applied to measure the difference between sub-genotypes of rs659366 (*P* = 0.017) and rs660339 (*P* = 0.026), followed by *post hoc* multiple comparisons via the LSD method of which *P* values were presented in the figure.

We observed a mean difference between genotypes rs659366 and rs660339 with respect to mean LTL (*P* = 0.017 and *P* = 0.026, respectively, ANOVA) (Fig. 1). This difference remained significant after adjustments for all known cardiovascular risk factors in the General Linear Models (including CVD risk that did not demonstrate significant interactions initially) (Table 3). Adjustments were made for age and gender (Table 3). The mean relative LTL of rs659366 GG genotype carrier is 1.257 ± 0.186. We also note for rs659366 A allele a corresponding increase in LTL. For example, the mean LTL of one A allele carriers is 1.288 ± 0.230 and of two A alleles carriers is 1.314 ± 0.253. However, by comparison with the rs660339 CC genotype, CT or TT genotypes result in a longer LTL of similar size, for example, with a mean LTL of 1.254 ± 0.187, 1.292 ± 0.229 and 1.297 ± 0.242, respectively. When we combine the CT and TT genotypes, rs660339 T allele carriers (CT/TT genotype) have an increased mean relative LTL of approximately 0.04, compared to non-T allele carriers (CC genotype; *P* = 0.004).

## Discussion

Our study findings demonstrate that the *UCP2* rs659366 A allele is associated with longer LTL in a rural population without diabetes or pre-diabetes. Furthermore, we have shown that rs659366 AA genotype carriers are associated with longer relative mean LTL (1.314 ± 0.253) in comparison to GA (1.288 ± 0.230) and GG (1.257 ± 0.186) genotype carriers. This finding is not surprising as the *UCP2* gene rs659366 is located in the *UCP2* promoter region, and includes a putative *UCP2* transcription initiation site that regulates *UCP2* mRNA expression levels^20^. A simplistic physiological interpretation of these results is that the A allele rs659366 favourably increases functional cellular mitochondrial *UCP2* expression. This in turn may reduce oxidative stress via mild mitochondrial uncoupling and in doing so protects telomeres from shortening during periods of oxidative stress.

In contrast to our data, Salpea *et al*. (2011) found that rs659366 A allele carriers had shorter LTL than GG genotype carriers^30^. However, also in contrast to our own study, this study was conducted in Caucasian type 2 diabetes patients. In our current study on 950 participants free from diabetes and pre-diabetes, longer LTL is associated significantly with AA and GA genotype, in comparison to GG. Notably, accumulated evidence has provided strong support for a significant direct association between telomere shortening and type 2 diabetes^32^,^33^,^34^,^35^. Consequently, the presence of diabetes within the Salpea *et al*. study group would act as a strong confounding factor when examining the association between this SNP and telomere length. Thus, it is reasonable to hypothesize our findings are the opposite to the report of Salpea *et al*. due to the difference in type 2 diabetes status of participants in the two studies.

Our data also show that participants carrying rs660339 TT or CT genotype have longer LTL (1.297 ± 0.242 for TT and 1.292 ± 0.229 for CT) than the CC genotype (1.254 ± 0.187), suggesting that the T allele is associated with longer LTL. The rs660339 UCP2 gene causes a single amino acid change at position 55 of the UCP2 protein. Currently, there are no studies that demonstrate any functional and/or expression change in UCP2 directly associated with this specific single amino acid change^36^. A possible explanation is that rs660339 acts as a tag SNP, with a corresponding region being a haplotype block (or haploblock) containing related UCP2 SNPs such as rs659366. For example, the rs659366 lies in a 17-kb block covering the entire gene and its flanking sequences and linkage disequilibrium remains high for the more distal rs660339 SNP (D′ = 1.0; r^2^ = 0.83) 37. This suggests that rs659366 is a more informative SNP for the entire *UCP2* gene.

In our study, the ratio of LTLs for two homozygous genotypes (longest to shortest) for UCP2 SNP rs660339 is 1.03 and rs659366 is 1.05, that are closely related, which suggests the possibility for a block gene tag area. Additionally, rs659366 has the larger mean difference of LTL across sub-genotypes (difference = 0.057) compared to that of rs660339 (difference = 0.043), consistent with rs659366 being the functional variant driving LTL.

Shortened telomeres are believed to contribute to beta-cell dysfunction^6^ and data confirms an association between shortened telomeres and T2DM^38^,^39^. Our data show an association between the rs659366 SNP and telomere length, suggesting that the rs659366 SNP should also show an association with T2DM. However, a clear association between the rs659366 SNP and T2DM remains unclear^40^. Whether there is some interaction between *UCP2* gene-defined risk and risk of diabetes requires some further investigation. On the other hand, some studies have reported that rs660339 is a T2DM susceptibility locus for Asian populations, but not in populations with European descent^40^.

It could be argued that the rs659336 SNP (−866G/G genotype) may be a risk factor for diabetes and therefore could be associated with shorter telomeres in our population. Bulotta *et al*. reported a 12% associative risk of T2DM with the −866G/G genotype^37^. Indeed, we found an association with the −866G/G genotype and short telomeres. On the other hand, based on absolute risk of 12% of developing diabetes due to a −866G/G genotype (using the associative risk from the Bulotta *et al*. study), only 47/390 subjects would be theoretically at risk of developing T2DM. Hence, the risk of diabetes would be considered low. Thus, pre-existing shortened telomeres due to this pre-determined diabetes is unlikely. Thus, we suggest that diabetes risk due to a −866G/G genotype is unlikely to influence our primary associations and models with LTL and *UCP2* genotypes.

Within the current study, we also find shorter LTL is associated with a higher level of triglycerides (coefficient = −0.016, *P *= 0.041). Previous studies have demonstrated a mixed relationship between high triglyceride levels and shorter telomeres, with some studies demonstrating a significant relationship^41^,^42^, whereas other studies have not^43^,^44^. Interestingly, in a study of subjects with diabetes, the presence of the GG genotype of the -866G/A (rs659366) is associated with higher triglycerides (≥1.70 mM), total cholesterol (≥6.0 mM) and LDL-cholesterol (≥3.35 mM) levels in T2DM patients^45^. In our samples we observed no significant interaction between *UCP2* rs659366 and lipids. However, a weak but significant interaction between the rs660339 SNP and triglyceride levels was observed in our data. Of note, our data did not observe an association between LTL and the cardiovascular risk factors of gender, smoking and BMI. While these associations have been frequently observed previously^46^,^47^,^48^, other well powered studies have also failed to observe statistically significant associations between these risk factors and LTL^49^,^50^,^51^,^52^. A possible explanation for this study not observing these associations is our modest sample size.

Our study also addresses perceived limitations, such as causality cannot be inferred from association studies *per se*. The subjects’ rural environment is unique to our population. Our findings would need to be replicated in a more heterogeneous population to extend these findings. We note that the rs660339 distribution frequency in our samples deviates from the Hardy-Weinberg equilibrium. This finding was not due to technical error, for example, when based on a 100 percent concordance of a 1% random selected validation sampling of the genotyping results, the rs660339 was determined to have been genotyped successfully. A possible explanation for the observed deviation from the Hardy-Weinberg equilibrium is that we may have artificially induced genetic drift by restricting our population to individuals with neither diabetes or pre-diabetes (with their greater likelihood of insulin resistance), while considering a gene (rs660339) that may be causally associated with insulin resistance^53^.

It is well known that telomere length is regulated and influenced by many gene pathways, physiological networks and biochemical pathways^54^,^55^,^56^. Previously an interaction between UCP2 SNPs and telomere length in diabetes patients has been reported^30^. In our study, by excluding those with diabetes and pre-diabetes, we have removed disease-related background levels of chronic inflammation and oxidative stress that could theoretically induce shortening of telomere length. We demonstrate that *UCP2* rs659366 A allele and rs660339 T allele are both related to longer LTL in subjects without diabetes, independent of cardiovascular risk factors. We thus have a better understanding of the direct interactions between *UCP2* genotypes and telomere length.

## Methods

### Participants and study design

A hypothesis generating study, UCP2 genotypes were predetermined to be tested in a non-diabetic population. Using the Crossroad Study data base containing clinical phenotypic data^57^, we selected all 950 participants who had neither diabetes nor pre-diabetes (impaired glucose tolerance or impaired fasting glucose) and where corresponding whole blood samples were available for DNA extraction to assess *UCP2* genotypes and telomere length. All 950 participants had a fasting glucose of ≤5.5 mmol/L, random glucose ≤7.7 mmol/L and HbA1c ≤5.6 mmol/L (American Diabetes Association criteria^58^).

Subjects from the data base had clinical information collected between June 2001 and March 2003 and were residents of Shepparton-Mooroopna and major surrounding towns in Victoria, Australia^57^. Historically the data base was constructed via a two-step process: interviews conducted with all residents in 2376 randomly selected households (half in the regional centre) with a 70% response rate, and then invitations were given out for all usual residents (resident in the area for at least 6 months) aged ≥25 years to attend for a ‘clinic’ assessment and blood bio-banking with a 61% attendance rate.

Cardiovascular risk measures that were included in models: Blood pressure was measured three times using a standardized method and the mean of the two closest measurements recorded. Height was measured without shoes using a stadiometer and weight was measured without shoes and excess clothing. BMI was calculated as body mass divided by square of height (kg/m^2^). Waist circumference was measured at the level halfway between the lowest ribs and the iliac crest. Glucose, lipids and HbA1c level were measured as previously reported^59^.

### Measurement of DNA

Genomic DNA was extracted from each participant’s peripheral blood sample using a QIAamp DNA Blood Mini Kit (Cat No.: 51106, QIAGEN, Venlo, Netherlands). DNA binds specifically to the QIAamp silica-gel membrane and was dissolved in 100 μL DNase-free water. DNA concentration was tested using a Nanodrop 1000 (Thermo Scientific, MA, U.S.). DNA was stored at −30 °C until usage. Two UCP2 SNPs were genotyped: rs659366 (Chromosome 11, 73983709 (Assembly GRCh38.p2)) and rs660339 (Chromosome 11, 73978059 (Assembly GRCh38.p2)). The two *UCP2* SNPs were genotyped on genomic DNA extracted from 950 peripheral blood samples using Taqman SNP Genotyping Assay (rs659366: C_8760350_10, rs6690339: C_903746_1, Life Technologies, CA, U.S.), performed on a LightCycler 480 (Roche, Penzberg, Germany). The standard 5 μL PCR reaction system (including 15 ng of genomic DNA template) was prepared using TaqMan Universal PCR Master Mix reagent kits under the guidelines provided. 1% of samples are replicated as genotyping quality control and results maintained 100% concordance. Mean relative LTL of each samples were measured using monochrome multiplex quantitative PCR method with 15 ng of genomic DNA as template^60^. Briefly, a relative measurement of the amplification of telomeric DNA sequences (T) normalised by single copy gene (S, albumin gene) was produced within each reaction well, in comparison to a common serial diluted reference DNA sample spanning 3.75-60 ng/μL. All samples were analysed in triplicate and the mean of the three T/S ratios was recorded. Multiplex quantitative PCR was performed using QuantStudio 12K Flex System (Life Technologies, CA, U.S.). The overall average coefficient of variation (CV, standard deviation divided by mean) for all the samples measured in triplicate was 3.24%.

### Statistics

All values are expressed as mean ± standard deviation when describing quantitative variables, unless otherwise indicated. The ANOVA test was used to compare quantitative variables between subgroups and the Chi-square test for categorical variables. The relationship between telomere length and diabetes risk factors and/or SNPs was evaluated using a General Linear Model. All data analysis was performed using SPSS 22.0 (IBM, Armonk, New York, U.S.). A two-sided *P*-value <0.05 was considered statistically significant.

## Additional Information

**How to cite this article**: Zhou, Y. *et al*. Interactions between UCP2 SNPs and telomere length exist in the absence of diabetes or pre-diabetes. *Sci. Rep.* **6**, 33147; doi: 10.1038/srep33147 (2016).

## Figures and Tables

**Table 1:** Characteristics of participants.

	overall	rs659366			*P*	rs660339			*P*
		AA	GA	GG		TT	CT	CC	
participants, n	950	138	422	390		183	413	347	
age, years†	50.2 ± 15.2	51.0 ± 15.4	50.2 ± 15.4	49.9 ± 14.8	0.735	51.2 ± 15.4	49.9 ± 15.3	50.0 ± 14.9	0.590
women, %*	59.6	63.0	57.8	60.3	0.521	63.3	58.1	59.1	0.471
smokers, %*	17.2	11.6	18.2	17.9	0.171	14.2	17.2	18.4	0.466
BMI, kg/m^2^	27.1 ± 5.0	27.2 ± 4.8	26.7 ± 4.8	27.4 ± 5.4	0.204	27.1 ± 4.9	26.8 ± 4.7	27.3 ± 5.4	0.379
waist, cm	91.9 ± 13.5	91.6 ± 12.1	91.3 ± 12.7	92.6 ± 14.7	0.397	91.2 ± 12.4	91.5 ± 12.6	92.7 ± 14.9	0.354
SBP, mmHg	128.2 ± 21.2	127.5 ± 22.4	127.5 ± 21.0	129.3 ± 21.2	0.457	127.4 ± 22.8	128.0 ± 20.7	129.0 ± 21.1	0.686
DBP, mmHg	71.2 ± 9.9	70.4 ± 10.5	70.1 ± 9.3	72.0 ± 10.2	0.112	70.5 ± 10.4	70.9 ± 9.3	71.9 ± 10.3	0.187
cholesterol, mmol/L	5.31 ± 0.98	5.34 ± 1.02	5.29 ± 1.01	5.31 ± 0.94	0.854	5.37 ± 1.06	5.27 ± 0.98	5.31 ± 0.94	0.570
HDL-C, mmol/L	1.48 ± 0.38	1.48 ± 0.36	1.48 ± 0.36	1.47 ± 0.41	0.976	1.47 ± 0.35	1.49 ± 0.37	1.45 ± 0.40	0.264
triglyceride, mmol/L	1.35 ± 1.03	1.42 ± 1.08	1.34 ± 1.19	1.33 ± 0.81	0.645	1.52 ± 1.74	1.27 ± 0.71	1.35 ± 0.83	0.028
HbA1c, %	5.11 ± 0.22	5.09 ± 0.22	5.10 ± 0.22	5.11 ± 0.22	0.470	5.10 ± 0.22	5.10 ± 0.22	5.12 ± 0.22	0.608

^§^*P* value of ANOVA to compare quantitative variables or the Chi-square test for categorical variables between genotype subgroups.

^†^All values expressed as mean ± standard deviation when describing quantitative variables, unless otherwise indicated. BMI, body mass index; SBP, systolic blood pressure; DBP, diastolic blood pressure; HDL-C, high density lipoproteins-cholesterol; HbA1c, glycated haemoglobin.

^*^Percentage.

**Table 2:** Relationship between mean telomere length and CVD risk factors.

	Coefficient*	95% CI	Std. Error	*P*
age	−0.004	(−0.005, −0.003)	0.001	<0.001
gender	−0.016	(−0.055, 0.023)	−0.020	0.431
smokers	−0.028	(−0.064, 0.009)	0.019	0.138
BMI	−0.001	(−0.007, 0.005)	0.003	0.819
waist	<0.001	(−0.002, −0.003)	0.001	0.927
SBP	−3.964E−05	(−0.001, 0.001)	<0.001	0.933
DBP	<0.001	(−0.002, 0.002)	0.001	0.784
cholesterol	0.010	(−0.006, 0.026)	0.008	0.219
HDL−C	0.024	(−0.021, 0.068)	0.023	0.300
triglyceride	−0.016	(−0.031, −0.001)	0.008	0.041
HbA1c	0.004	(−0.062, 0.069)	0.033	0.911

^*^Relationship was evaluated using General Linear Model without adjustment.

**Table 3:** Relationship between mean telomere length and UCP2 SNPs polymorphism.

	TL ± Std. Deviation	Coefficient*	95% CI	Std. Error	*P*
rs659366					
AA	1.314 ± 0.253	0.065	(0.024, 0.105)	0.021	0.002
GA	1.288 ± 0.230	0.033	(0.005, 0.062)	0.015	0.022
GG	1.257 ± 0.186	0			
AA/GA	1.295 ± 0.236	0.041	(0.014, 0.068)	0.014	0.003
GG	1.257 ± 0.186	0			
rs660339					
TT	1.297 ± 0.242	0.051	(0.014, 0.089)	0.019	0.007
CT	1.292 ± 0.229	0.037	(0.007, 0.066)	0.015	0.016
CC	1.254 ± 0.187	0			
TT/CT	1.294 ± 0.233	0.041	(0.014, 0.069)	0.014	0.004
CC	1.254 ± 0.187	0			

^*^Relationship was evaluated using General Lineal Model, and adjusted by age, gender, smokers, BMI, waist, SBP, DBP, cholesterol, HDL, triglyceride, HbA1c.
